# Pregnancy-related aeromedical retrievals in rural and remote Australia: national evidence from the Royal Flying Doctor Service

**DOI:** 10.1186/s12913-021-06404-5

**Published:** 2021-04-26

**Authors:** David Gonzalez-Chica, Marianne Gillam, Susan Williams, Pritish Sharma, Matthew Leach, Martin Jones, Lucie Walters, Fergus Gardiner

**Affiliations:** 1 Adelaide Rural Clinical School, The University of Adelaide, Helen Mayo North building, 109 Frome Road, Level 1, Room 106, Adelaide, SA 5005 Australia; 2 Department of Rural Health, University of South Australia, Mt Barker, SA Australia; 3 Royal Flying Doctor Service of Australia, Canberra, ACT Australia; 4 National Centre for Naturopathic Medicine, Southern Cross University, East Lismore, NSW Australia

**Keywords:** Rural population, Maternal health services, Pregnancy complications, Emergency medical services, Air ambulances

## Abstract

**Background:**

Inequalities in the availability of maternity health services in rural Australia have been documented, but not the impact on aeromedical retrievals. This study aims to examine the prevalence of pregnancy-related aeromedical retrievals, the most common conditions (overall and in specific age groups), and their distribution according to operation area and demographic characteristics.

**Methods:**

Cross-sectional study using administrative data from the Royal Flying Doctors Service (RFDS) including all pregnant women aged 15–49 years retrieved by the RFDS between 2015 and 2019. All pregnancy-related aeromedical retrievals were classified according to the International Classification of Diseases, Tenth Revision (ICD-10, chapter XV). The distribution of pregnancy-related conditions was presented overall and stratified by age group (i.e. < 20 years, 20–34 years and 35+ years). Retrieval and receiving sites were geographically mapped with Tableau mapping software® based on postcode numbers of origin and destination.

**Results:**

A total of 4653 pregnancy-related retrievals were identified (mean age 27.8 ± 6.1 years), representing 3.1% of all RFDS transfers between 2015 and 18 and 3.5% in 2018–19 (*p*-value 0.01). The highest proportion of pregnancy-related retrievals (4.8%) occurred in Western operation. There was an apparent increase in pregnancy-related retrievals in South Australia and the Northern Territory (Central Operation) in 2018–19. Preterm labour/delivery was responsible for 36.4% of all retrievals (40.7% among women aged 15–19 years) and premature rupture of membranes for 14.9% (19.4% among women aged 35–49 years). Inter-hospital transfers represented 87.9% of all retrievals, with most patients relocated from rural and remote regions to urban hospitals; most retrievals occurred during the day, with a median distance of 300 km. Adolescents and Aboriginal and Torres Strait Islander were overrepresented in the sample (four and eight times higher than their metropolitan counterparts, respectively).

**Conclusions:**

The proportion of pregnancy-related aeromedical retrievals varies geographically across Australia. Overall, one-third of retrievals were related to preterm/delivery complications, especially among adolescents. Most retrievals performed by the RFDS are susceptible to public health strategies aimed at improving antenatal care and preventing unintended pregnancies among adolescents and Aboriginal and Torres Strait Islander women. Greater capacity to manage pregnancy conditions in rural hospitals could reduce the requirement for aeromedical inter-hospital transfers.

## Background

Australia is a large country with vast, sparsely populated areas. Of the 25 million inhabitants in Australia, 72% live in major cities and 18% in inner-regional areas, with the remainder residing in outer-regional (8.2%), remote (1.2%), and very remote areas (0.1%) [1]. People living in small communities in the remote interior of Australia are often socially isolated, more exposed to lifestyle determinants of illness, and have limited access to health services. There are also disparities in the geographic distribution of health conditions, with a higher prevalence of diabetes, hypertension, and renal disease in rural or remote communities compared to those living in major cities [2–4].

Pregnancy-related conditions and behaviours present a different geographic distribution across urban and rural Australia. Women living in rural and remote Australia have a fertility rate 30% higher than those from major cities, are up to 4.7 times more likely to smoke during pregnancy, and 62% more likely to die during pregnancy or childbirth [5–9]. A combination of access inequalities to maternity services and higher prevalence rates of chronic disease in remote and very remote areas contribute to poorer maternal outcomes, including a higher prevalence of gestational hypertension, preeclampsia, and maternal mortality [6, 8–11]. These same factors correlate with higher frequencies of preventable adverse consequences for the newborn [5, 8–11]. In 2018, the prevalence of low birth weight in very remote Australia was almost twice that of major cities (11.2% vs. 6.6%, respectively), while the need of special care nursery or neonatal intensive care units was 37% more prevalent [5]. Intrauterine growth restriction, prematurity, stillbirths and neonatal mortality are also more prevalent in rural and remote Australia [9–11].

Despite the fact that these disparities have been largely reported, inequalities in the distribution of maternity services persist [5, 6, 8, 12]. Rural and remote Australian communities are less likely to have maternity services, and many patients are required to travel hundreds of kilometres to access health care [6, 12, 13]. Accordingly, aeromedical transfers of women with pregnancy-related complications from rural and remote communities to tertiary care facilities are often required [13, 14]. Notwithstanding, few studies have investigated the frequency of aeromedical retrievals in Australia; further, these studies have been limited to the analysis of specific pregnancy-related conditions or have a limited geographical range [15–18]. Therefore, there is a lack of understanding of the specific pregnancy, childbirth, and puerperium conditions requiring aeromedical retrievals at a national level, and their distribution according to area and demographic characteristics.

The Royal Flying Doctor Service (RFDS) is the world’s largest aeromedical organisation that provides health care services for people in rural or remote Australia who cannot access traditional services. Other aeromedical providers in the country are largely state-based retrieval services [3, 13, 14, 19]. Data from the RFDS have been mainly used to explore retrieval of trauma patients or airway management, with a preponderance of studies using a cross-sectional design [14]. More recent studies exploring national RFDS retrieval activity have focused on chronic conditions such as stroke, chronic kidney disease, and mental health [20–22]. A recent study used RFDS data (2015–2017) to examine aeromedical retrievals and outcomes for a cohort of women with pregnancy-related conditions [18].

Insights into the main pregnancy-related conditions requiring aeromedical retrievals may help support future health workforce development and health services planning to address inequalities in maternity health care in rural and remote Australia. To address this need, this study aimed to identify: 1) the prevalence of pregnancy-related aeromedical retrievals by operation area (including the demographic features of patients) between 2015 and 2019, 2) the most common pregnancy-related conditions (overall, by operation area and in specific age groups) requiring aeromedical retrieval, and 3) the main transfer sites (i.e. location of flight departures and destinations) for pregnant women transported by the RFDS.

## Methods

### Study design

This study is a retrospective analysis using administrative de-identified data previously collected by the RFDS.

### Setting

This study uses data from the RFDS clinical and aeromedical databases, including data of all pregnancy-related aeromedical retrievals performed by the RFDS in Australia between 2015 and 2019. More than 30,000 aeromedical retrievals are undertaken by the RFDS annually across Australia [19]. The databases comprise patient demographic and clinical data, as well as in-flight information, on every patient that has received care through RFDS as part of any aeromedical retrieval, as per government reporting requirements. These data are routinely collected by in-flight RFDS staff using either paper-based or electronic methods. While patient data collection forms and systems differ between the four RFDS operation areas in Australia [Central (North Territory and South Australia), Queensland, South East (New South Wales) and Western Operations (Western Australia)], all areas collect data for the same variables. Data from paper-based records are entered into an electronic medical record by trained administrative RFDS staff following a standard protocol. The same staff also code patient diagnoses based on the International Statistical Classification of Diseases and Related Health Problems, tenth edition [ICD-10] categories, and all coding is verified by public health and finance managers [23].

### Sample

This study included data on all women aged 15–49 years who were retrieved by air between 1st July 2015 and 30th June 2019 by the RFDS from anywhere in rural or remote mainland Australia (i.e. excluding Tasmania) for the management of pregnancy-related conditions (ICD-10, chapter XV Pregnancy, childbirth and the puerperium) [23]. The Australian Statistical Geography Standard (ASGS) defines five classes of relative remoteness across Australia (major cities, inner regional, outer regional, remote or very remote) based on the population size of the locations and distance from service centres [24]. In this paper, we use the term rural to refer to outer regional, and remote to refer to remote or very remote areas. Only data related to primary evacuations (PE, emergency medical service provided to people who are in a potentially life-threatening condition and are beyond the standard medical infrastructure) and interhospital transfers (IHT, transfer of patients from a rural or remote hospital to a hospital in an urban centre, or from a lower to a higher level of care hospital in rural or remote locations) were included [13]. Therefore, a rural town can be both, a retrieval source and a retrieval destination. Electronic records related to patient repatriations (i.e. transfer of patients from tertiary hospitals to the patient’s rural or remote place of residence) were excluded from the analysis. The latter represents 1.4% (*n* = 2055) of all RFDS retrievals for any cause during the investigated period (2 repatriation cases of pregnancy-related retrievals).

### Data preparation and analysis

De-identified electronic records were extracted using Alteryx® and then normalised for data analysis. De-identified patient-level data obtained from the electronic records included age (in years), aeromedical retrieval diagnosis (ICD-10 three-character categories) [23], and self-reported Aboriginal or Torres Strait Islander (yes, no, non-stated) status. Flight information was also extracted, including departure and destination locations (i.e. location, airport, hospital), recorded flight distance, time of the day and operation area. Multiple transfer legs for the same patient were combined as single episodes of care for analysis.

Descriptive summary statistics were used to report the data. Categorical variables are presented as percentages and continuous variables as means and standard deviations. Maternal age distribution of the RFDS aeromedical retrievals was compared to Australian benchmarks using data from the Australian Institute of Health and Welfare (AIHW) [5].

Results for the total number of retrievals, and distribution according to the financial year (2015–16, 2016–17, 2017–18, 2018–19), maternal age and indigenous status were stratified by operation area. The distribution of pregnancy-related conditions was presented overall, per operation centre and age group (i.e. < 20 years, 20–34 years and 35+ years). The presentation of the results per age group was performed because childbirth in adolescence or among women aged 35+ years increases the risk of maternal morbimortality [25, 26]. Differences in the patient distribution according to the operation area or age groups were assessed using the chi-square test (categorical variables) or ANOVA (continuous variables). All statistical analyses were conducted in STATA 15.0 (StataCorp, Texas, USA). Retrieval and receiving sites were geographically mapped with Tableau mapping software® based on the postcode numbers of origin and destination.

### Ethics approval

As this project uses de-identified records of routine data previously collected by the RFDS, specific patient consent forms were not required. The protocol of the study and waive of consent were approved by the Human Research Ethics Committee of the University of South Australia (Application ID: 202236) and the RFDS Clinical and Health Services Research Committee, which provides oversight for RFDS research projects (Application ID: 201904091).

## Results

**Fig. 1:**
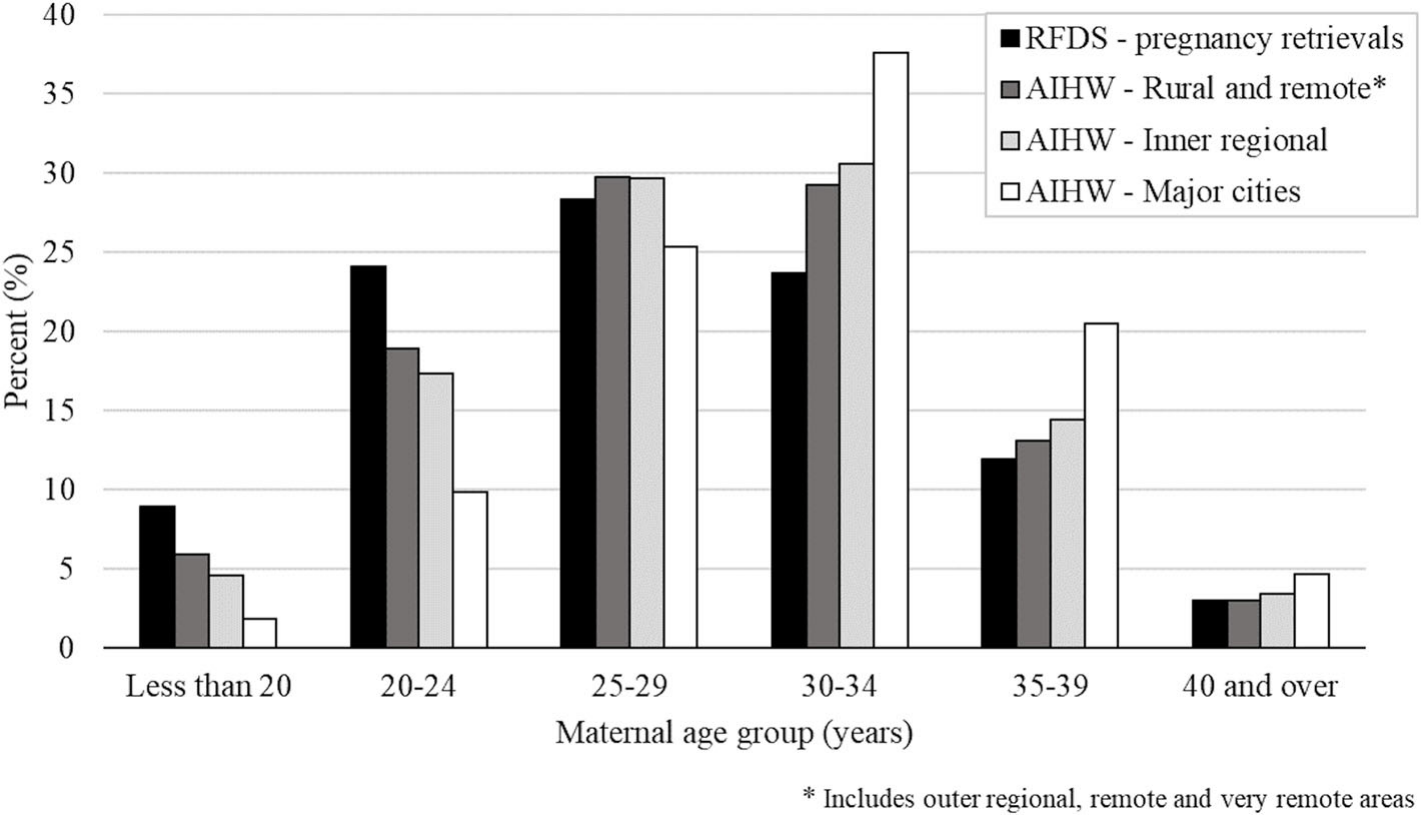
Maternal age of pregnancy-related retrievals by the Royal Flying Doctor Service (2015–2019, *N* = 4653) compared to maternal age distribution in Australia according to data from the Australian Institute of Health and Welfare [5]

RFDS performed 147,979 aeromedical retrievals (PEs and IHTs) between 2015 and 2019. Of these, 4653 (3.1%) were pregnancy-related retrievals (mean age 27.8 ± 6.1 years). The age distribution of women requiring an RFDS pregnancy-related retrieval was lower than the maternal age profile of women from urban settings in Australia (Fig. 1) [5].

Pregnancy-related aeromedical retrievals were lower in the South East (1.2% of all retrievals) and higher in the Western operation area (4.8%; *p*-value < 0.001) (Table 1). There was a slightly higher proportion of pregnancy-related transfers in 2018–19 (3.5%) compared to previous years (~ 3.1%, *p*-value 0.01), which was related to a higher number of retrievals from Central operation in that year. Pregnancy-related retrievals from Queensland were also slightly higher in the last two financial years, while less of these transfers occurred in the South East operation in the same period. A U-shaped pattern was observed in the Western operation. The average maternal age of retrieved patients was similar in all operation areas. The proportion recorded as Aboriginal or Torres Strait Islander peoples was similar in Central, South East and Western operations (39–42%) and lower in Queensland, although the last area included almost all cases where the Aboriginality was not stated.

**Table 1:** Summary statistics for pregnancy-related transfers in Australia by operation area, Royal Flying Doctor Service, 2015–2019

		Total	Operation area				***p***-value
			Centra (NT – SA)	Queenslan (QLD)	South East (NSW)	Western (WA)	
	**All**^**a**^	147,979	37,049	44,874	32,358	33,666	
	**Pregnancy** ^**a,b**^	4653	1125	1521	393	1614	
	%	*3.1%*	*3.0%*	*3.4%*	*1.2%*	*4.8%*	< 0.001*
**Years**							
2015–16	**All**^**a**^	35,949	8758	10,856	7861	8457	< 0.001*
	**Pregnancy** ^**a,b**^	1104	249	299	112	444	
	%	*3.1%*	*2.8%*	*2.8%*	*1.4%*	*5.3%*	
2016–17	**All**^**a**^	36,567	9103	11,221	8023	8218	< 0.001*
	**Pregnancy** ^**a,b**^	1133	252	371	120	390	
	%	*3.1%*	*2.8%*	*3.3%*	*1.5%*	*4.7%*	
2017–18	**All**^**a**^	37,472	9686	11,249	8106	8428	< 0.001*
	**Pregnancy** ^**a,b**^	1106	244	435	65	362	
	%	*3.0%*	*2.5%*	*3.9%*	*0.8%*	*4.3%*	
2018–19	**All**^**a**^	37,991	9502	11,548	8368	8563	< 0.001*
	**Pregnancy** ^**a,b**^	1310	380	416	96	418	
	%	*3.5%*	*4.0%*	*3.6%*	*1.1%*	*4.9%*	
	*p*-value	0.01^c^	< 0.001^c^	< 0.001^c^	< 0.001^c^	< 0.001^c^	
**Women characteristics**							
**Age in years**	***Mean ± SD***	*27.8 ± 6.1*	*27.4 ± 6.1*	*27.7 ± 6.0*	*26.6 ± 6.2*	*28.3 ± 6.3*	< 0.07**
**Aboriginal or Torres Strait Islander**							
Yes	***n*** **(%)**	1584 (34.0)	446 (39.6)	347 (22.8)	166 (42.2)	625 (38.7)	< 0.001†
No		2672 (57.4)	678 (60.3)	786 (51.7)	223 (56.7)	985 (61.0)	
Not stated		397 (8.5)	1 (0.1)	388 (25.5)	4 (1.0)	4 (0.3)	
**Travel characteristics**							
**Distance flown (*n*** **= 1132)**	**Median [p25-p75]**	300 [211–464]	246 [213–347]	313 [232–472]	300 [239–380]	357 [196–564]	< 0.001††
**Flight authorisation times (*n*** **= 3343)**							
0 am - 6 am	***n*** **(%)**	369 (11.0)	114 (15.3)	102 (9.2)	29 (9.8)	124 (10.4)	< 0.001†
7 am - 7 pm		2353 (70.4)	478 (64.2)	832 (75.3)	213 (71.7)	830 (69.4)	
8 pm - 11 pm		621 (18.6)	153 (20.5)	171 (15.5)	55 (18.5)	242 (20.2)	

*NT* North Territory, *SA* South Australia, *QLD* Queensland, *NSW* New South Wales, *WA* Western Australia

a – Excluding repatriations

b – Females aged 15–49 years with an obstetric condition (ICD10) as the primary diagnosis for retrieval. Multiple transfer legs considered as single transfers

c – *P*-value for the comparison of the proportion of pregnancy-related retrievals between 2015 and 16 and 2018–19

* Chi-squared test of heterogeneity comparing the prevalence of pregnancy-related retrievals between operation areas

** ANOVA test for heterogeneity for the difference in maternal age according to the operation area

† Chi-squared test for heterogeneity for the distribution of indigenous status or flight authorisation times according to operation area

†† Kruskal-Wallis test for the difference in median distance flown according to the operation area

Table 1 also shows the median distance flown (available for 1132 retrievals only) was 300 km [interquartile range (p25-p75) 211–464 km] and the longest 1815 km (data not shown in table). The distance flown in Western operation was greater than in other areas. Flight authorisation times collected for 3343 patients showed that 70.4% of retrievals occurred between 7 am and 7 pm. A higher proportion occurred overnight in Central operation (35.8%) than in other areas (~ 25–30%).

### Transfer origins and destinations

**Fig. 2:**
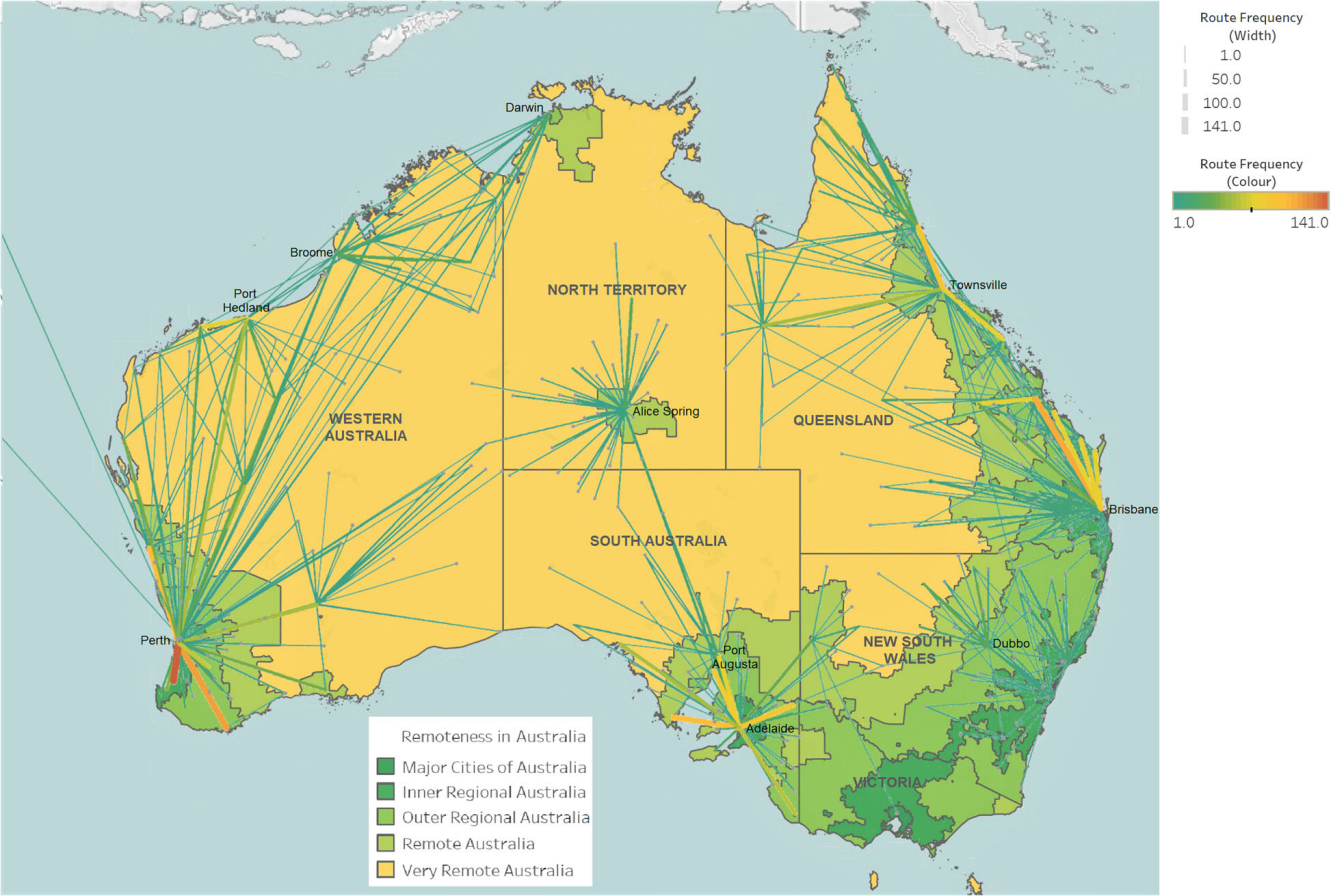
Pregnancy-related transfers in Australia performed by the Royal Flying Doctor Service between July 1st 2015 and June 30th 2019 (*n* = 4653) and remoteness of location in Australia [24]. The spider map details the pick-up, transfer location and route frequency of the aeromedical retrievals

Most patient transfers originated in rural and remote regions and ended in inner-regional or major cities (Fig. 2), with IHTs representing 87.9% of all pregnancy-related retrievals. There were two main receiving sites in Northern Territory (Darwin and Alice Springs) and South Australia (Adelaide and Port Augusta), compared to multiple sites in other states. The leading receiving sites per operation area were: Adelaide (60.6%) and Alice Springs (26.2%) in Central (*n* = 1125); Brisbane (34.4%) and Townsville (21.8%) in Queensland (*n* = 1521); Dubbo (31.0%) in South East (*n* = 393); and Perth (52.3%), Port Hedland (9.5%) and Broome (9.5%) in Western operation (*n* = 1614).

### Diagnoses: overall

Overall, 95.4% of patients had data on the specific pregnancy-related diagnosis (Table 2). The most frequent ICD-10 diagnoses by block were ‘Complications of labour and delivery’ (O60-O75; 41.5% with a similar prevalence in all operation areas), ‘Maternal care related to the foetus and amniotic cavity and possible delivery problems’ (O30-O48; 29.4% with a higher prevalence in South East and Western areas), and ‘Oedema, proteinuria and hypertensive disorders in pregnancy, childbirth and the puerperium’ (O10-O16; 9.5% with a similar prevalence in all operation areas). Pregnancy with abortive outcome (O00-O08) was more frequent in Queensland and Western areas, while retrievals related to normal term delivery (O80-O84) were practically absent in the last. In terms of specific diagnoses, the most frequent conditions overall were threatened preterm labour and delivery (O60; 36.4%); premature rupture of membranes (O42; 14.9%); antepartum haemorrhage (O46; 6.3%); and ectopic pregnancy (O00; 5.4%).

**Table 2:** ICD-10 diagnoses for pregnancy-related aeromedical retrievals* by the Royal Flying Doctor Service according to the operation area, 2015–2019 (*N* = 4438)

ICD-10 diagnoses*	***N*** (%)	Operation area				***p***-value**
		Central (NT – SA)	Queensland (QLD)	South East (NSW)	Western (WA)	
		%	%	%	%	
**Pregnancy with abortive outcome (O00-O08)**	**388 (8.7)**	**7.8**	**9.3**	**5.4**	**9.6**	**0.044**
*Ectopic pregnancy (O00)*	*240 (5.4)*	*4.1*	*7.0*	*3.7*	*5.4*	
*Complications following ectopic and molar pregnancy (O08)*	*9 (0.2)*	*0.4*	*0.4*	*0.0*	*0.0*	
*Others*	*139 (3.1)*	*3.4*	*2.0*	*1.7*	*4.2*	
**Supervision of high-risk pregnancy O09**	**27 (0.6)**	**0.0**	**2.0**	**0.0**	**0.0**	**< 0.001**
**Oedema, proteinuria and hypertensive disorders in pregnancy, childbirth and the puerperium (O10-O16)**	**421 (9.5)**	**10.0**	**8.1**	**8.9**	**10.5**	**0.15**
*Pre-existing hypertension (O10-O11)*	*25 (0.6)*	*1.2*	*0.7*	*0.9*	*0.0*	
*Gestational hypertension and preeclampsia (O13-O14)*	*356 (8.0)*	*7.6*	*6.2*	*7.4*	*9.9*	
*Eclampsia (O15)*	*20 (0.5)*	*0.6*	*0.7*	*0.3*	*0.2*	
*Others*	*20 (0.5)*	*0.5*	*0.5*	*0.3*	*0.4*	
**Other maternal disorders predominantly related to pregnancy (O20-O29)**	**240 (5.4)**	**8.7**	**5.4**	**6.3**	**2.9**	**< 0.001**
*Haemorrhage in early pregnancy (O20)*	*84 (1.9)*	*2.8*	*1.8*	*2.0*	*1.4*	
*Diabetes mellitus in pregnancy (O24)*	*34 (0.8)*	*1.8*	*0.6*	*0.3*	*0.3*	
*Other maternal care for other conditions related to pregnancy (O26)*	*56 (1.3)*	*2.1*	*1.3*	*2.6*	*0.4*	
*Abnormal findings on antenatal screening (O28)*	*34 (0.8)*	*0.9*	*1.6*	*0.0*	*0.2*	
*Others*	*32 (0.7)*	*1.2*	*0.2*	*1.4*	*0.7*	
**Maternal care related to the fetus and amniotic cavity and possible delivery problems (O30-O48)**	**1303 (29.4)**	**22.4**	**29.2**	**32.3**	**33.7**	**< 0.001**
*Premature rupture of membranes (O42)*	*663 (14.9)*	*10.8*	*14.4*	*15.7*	*18.1*	
*Antepartum haemorrhage (O46)*	*278 (6.3)*	*3.6*	*6.0*	*6.3*	*8.3*	
*Others*	*362 (8.2)*	*7.9*	*8.8*	*10.3*	*7.3*	
**Complications of labour and delivery (O60-O75)**	**1843 (41.5)**	**42.3**	**40.3**	**41.7**	**42.0**	**0.72**
*Threatened preterm labour and delivery (O60)*	*1616 (36.4)*	*34.3*	*34.8*	*34.9*	*39.5*	
*Others*	*227 (5.1)*	*8.0*	*5.4*	*6.9*	*2.5*	
**Normal term delivery (O80-O84)**	**115 (2.6)**	**5.0**	**3.4**	**3.4**	**0.1**	**< 0.001**
**Complications predominantly related to the puerperium (O85-O92)**	**49 (1.1)**	**1.6**	**1.0**	**1.1**	**0.9**	**0.31**
**Other obstetric conditions, not elsewhere classified (O94-O99)**	**52 (1.1)**	**2.2**	**1.3**	**0.9**	**0.4**	**< 0.001**
**Total retrievals with ICD-10 diagnoses – N**	**4438**	**1125**	**1349**	**350**	**1614**	

* Missing data for the three-character ICD-10 categories for 215 (4.6%) of the total 4653 pregnancy-related retrievals classified as Chapter XV

** Chi-squared test of heterogeneity comparing the prevalence of the three-character ICD-10 categories across operation areas

The distribution of pregnancy-related conditions by maternal age group was similar for most conditions (Table 3). However, retrievals related to complications of labour and delivery (O60-O75) were more prevalent among women aged 15–19 years, while ‘Maternal care related to the fetus and amniotic cavity and possible delivery problems’ (O30-O48) were more frequent among women aged 35–49 years.

**Table 3:** ICD-10 diagnoses for pregnancy-related aeromedical retrievals* by the Royal Flying Doctor Service according to age groups, 2015–2019 (*N* = 4438)

ICD-10 diagnoses*	Maternal age groups			***p***-value**
	15–19 years	20–34 years	35–49 years	
	%	%	%	
**Pregnancy with abortive outcome (O00-O08)**	**11.2**	**8.4**	**9.0**	**0.17**
*Ectopic pregnancy (O00)*	*6.8*	*5.4*	*4.9*	
*Complications following ectopic and molar pregnancy (O08)*	*0.0*	*0.2*	*0.3*	
*Others*	*4.4*	*2.8*	*3.9*	
**Supervision of high-risk pregnancy O09**	**0.8**	**0.6**	**0.6**	**0.90**
**Oedema, proteinuria and hypertensive disorders in pregnancy, childbirth and the puerperium (O10-O16)**	**9.7**	**9.1**	**11.6**	**0.13**
*Pre-existing hypertension (O10-O11)*	*0.5*	*0.4*	*1.6*	
*Gestational hypertension and preeclampsia (O13-O14)*	*8.4*	*7.9*	*8.4*	
*Eclampsia (O15)*	*0.3*	*0.5*	*0.4*	
*Others*	*0.5*	*0.3*	*1.0*	
**Other maternal disorders predominantly related to pregnancy (O20-O29)**	**3.9**	**5.6**	**5.3**	**0.39**
*Haemorrhage in early pregnancy (O20)*	*0.5*	*2.1*	*1.6*	
*Diabetes mellitus in pregnancy (O24)*	*0.3*	*0.6*	*1.8*	
*Other maternal care for other conditions related to pregnancy (O26)*	*1.0*	*1.2*	*1.5*	
*Abnormal findings on antenatal screening (O28)*	*0.3*	*0.9*	*0.4*	
*Others*	*1.8*	*0.7*	*0.0*	
**Maternal care related to the fetus and amniotic cavity and possible delivery problems (O30-O48)**	**20.4**	**28.6**	**38.4**	**< 0.001**
*Premature rupture of membranes (O42)*	*9.9*	*14.6*	*19.4*	
*Antepartum haemorrhage (O46)*	*3.9*	*6.1*	*8.3*	
*Others*	*6.5*	*7.8*	*10.7*	
**Complications of labour and delivery (O60-O75)**	**46.7**	**43.1**	**31.0**	**< 0.001**
*Threatened preterm labour and delivery (O60)*	*40.7*	*37.8*	*26.8*	
*Others*	*6.0*	*5.2*	*4.2*	
**Normal term delivery (O80-O84)**	**3.9**	**2.6**	**1.8**	**0.11**
**Complications predominantly related to the puerperium (O85-O92)**	**1.6**	**1.1**	**1.0**	**0.66**
**Other obstetric conditions, not elsewhere classified (O94-O99)**	**1.8**	**1.1**	**1.3**	**0.39**
**Total retrievals with ICD-10 diagnoses – N**	**383**	**3380**	**675**	

* Missing data for the three-character ICD-10 categories for 215 (4.6%) of the total 4653 pregnancy-related retrievals classified as Chapter XV

** Chi-squared test of heterogeneity comparing the prevalence of the three-character ICD-10 categories across operation areas

## Discussion

This is the first study investigating the distribution of pregnancy-related aeromedical retrievals across mainland Australia [14–18]. Four main findings can be drawn from the results. First, the proportion of pregnancy-related retrievals varied geographically, being highest in the Western area and lowest in the South East. While the total frequency of retrievals remained relatively steady over time, a larger number of transfers occurred in the Central operation in 2018–19. Second, nine out of 10 retrievals were IHT, most transfers occurred during the day, and the median travelled distance was 300 km. Third, threatened preterm labour/delivery and premature rupture of membranes represented half of all retrievals, but the former was more frequent among adolescents and the last among women aged 35–49 years. Finally, compared to their metropolitan counterparts, there was an overrepresentation of adolescents and Aboriginal or Torres Strait Islanders among those retrieved.

Transfer of patients over long distances is likely to be highly stressful for the women and their families, and costly for health services. Due to the large area size of Western Australia compared to other states, it is not surprising that the median distance travelled is also higher in that operation centre. Nonetheless, the higher proportion of pregnancy-related retrievals and the higher proportion of premature rupture of membranes in Western operation compared to other operation centres warrants further investigation. Innovative models of care are currently being considered to meet the needs of women within communities in rural and remote Western Australia [27]. Tracking retrievals over time would be a useful tool to evaluate the success of these strategies.

Overall, the higher number of pregnancy-related retrievals in 2018–19 is in contrast to the declining number of women from rural settings giving birth in Australia, which fall from 32,817 (of all in 2015 to 30,229 in 2018 (12.2 and 11.5% of all births, respectively). Moreover, 96% of all births in 2018 occurred in a hospital facility [5]. Most of these facilities are located in urban areas, [6, 8] so that even women with non-complicated pregnancies need to be moved away from their rural communities to give birth [9].

The rise of pregnancy-related aeromedical retrievals observed in Central operation in 2018–19 compared to previous years may suggest potential challenges in providing maternity care in rural and remote communities in that area, where only one-third of the 62 rural hospitals in South Australia have maternity services [28] and there are only two of these facilities in in rural settings of the Northern Territory [6]. According to a national study published in 2017, all 259 maternity health services in rural Australia were located in towns of 1000–25,000 inhabitants, and only 68% could perform C-sections. These services were primarily located in Victoria, New South Wales and Queensland, with few services available in the other states [6]. Moreover, not all rural maternity services with caesarean facilities have the neonatal care capability for infants born less than 37 weeks gestation [8, 9].

In our study, most transfers were related to early pregnancy (i.e. before 20 weeks gestation) or preterm (i.e. 20–36 weeks gestation) complications that were initially treated in a rural hospital before aeromedical retrieval. Similarly, Gardiner et al. [18] reported a high frequency of IHT pregnancy retrievals, with the primary reasons being preterm labour/delivery and premature rupture of membranes. Preterm hospitalisations and subsequent IHT are not infrequent. A study published in 2012 reported that 16% of pregnancies in New South Wales required a preterm hospitalisation, with 6% of these requiring IHT [29]. In consonance with our findings, IHT in that study were more frequent among women admitted for premature rupture of membranes (20%) or threatened preterm labour (12%). The high frequency of aeromedical IHTs indicates that many rural hospitals are not able to provide the level of support required for pregnancy complications. Interventions to increase rural capacity to manage these conditions could reduce the need for these transfers.

Adolescents and Aboriginal and Torres Strait Islander were overrepresented in the RFDS sample (four and eight times higher than metropolitan counterparts, respectively) [5, 9]. The overrepresentation of Aboriginal and Torres Strait Islander women in our study illustrates the increased risk of perinatal morbidity (e.g. 13.8% vs. 8.5% preterm birth rate among women who gave birth in 2018) and mortality (e.g. 13.8 vs. 6.6 deaths per 100,000) in this population relative to non-indigenous women [5, 9, 25]. These problems do not necessarily result from the younger age of Aboriginal and Torres Strait Islander mothers, but from factors such as socioeconomic disadvantage, higher frequency of co-morbidities, maldistribution of health services, and challenges faced by the health system to support these women [5, 8, 9, 30, 31].

A limitation of this study is the narrow range of sociodemographic and clinical characteristics reported in the RFDS database. The accuracy of the recorded working diagnosis made in flight is another potential limitation. However, the data coding process followed by the RFDS is considered the gold standard in aeromedical medicine [13, 14, 19, 21]. Moreover, less than 5% of the cases had missing data for the three-character ICD-10 categories.

## Conclusion

The proportion of pregnancy-related aeromedical retrievals varies geographically across Australia. Overall, one-third of the RFDS retrievals were related to preterm labour and delivery complications, and this was found to be more frequent among adolescents. These complications can be potentially reduced through appropriate antenatal care, identification and management of potential risk factors, and regular maternal follow-up. Greater capacity to manage these pregnancy conditions in rural hospitals could also reduce the requirement for aeromedical inter-hospital transfers. The implementation of effective, proactive, and culturally appropriate strategies aimed at improving antenatal care and preventing unintended pregnancies among adolescents and Aboriginal and Torres Strait Islander women is also warranted. Further insights are needed into the sociodemographic characteristics of retrievals, and the outcomes for mothers and babies. Such information may help inform more targeted research aimed at improving the prevention and management of pregnancy-related issues in rural and remote Australia.

## Data Availability

The data that support the findings of this study are available from the Royal Flying Doctor Service, but restrictions apply to the availability of these data, which were used under license for the current study, and so are not publicly available. Data are however available from the authors upon reasonable request and with permission of the Royal Flying Doctor Service.

## Abbreviations

AIHW: Australian Institute of Health and Welfare
ANOVA: Analysis of variance
GPS: Global Positioning System
ICD-10: International Statistical Classification of Diseases and Related Health Problems, tenth edition
IHT: Interhospital transfers
PE: Primary evacuations
p25-p75: Interquartile range
RFDS: Royal Flying Doctor Service
VIF: Variance inflation factor
